# Introduction to Public Health Surveillance Course

**Published:** 2013-02-22

CDC and the Rollins School of Public Health at Emory University will cosponsor the course, Introduction to Public Health Surveillance, May 20–24, 2013, at Emory University in Atlanta, Georgia. The course is designed for state and local public health professionals.

The course will provide theoretical and practical knowledge to design, implement, and evaluate effective public health surveillance programs. Topics scheduled for presentation include an overview and history of surveillance systems; planning considerations; sources and collection of data; analysis, interpretation, and communication of data; surveillance systems technology; ethics and legalities; state and local concerns; and future considerations. Tuition is charged.

Additional information and applications are available online (http://www.sph.emory.edu/epicourses); by e-mail ( pvaleri@emory.edu ); by mail (Emory University, Hubert Department of Global Health, 1518 Clifton Rd. NE, CNR Bldg., Rm. 7038, Atlanta, GA 30322); by telephone (404-727-3485); or by fax (404-727-4590).

